# Understanding pain in malignant mesothelioma: a prospective, longitudinal cohort study

**DOI:** 10.1186/s12904-026-02187-w

**Published:** 2026-06-13

**Authors:** Catriona R. Mayland, Michelle Collinson, Sam H. Ahmedzai, Joseph Lucas, Jacqui Gath, Liz Darlison, Duneesha Defonseka, Barry Laird, Helena Stanley, Sarah Danson, Clare Gardiner, Anna C. Bibby, the ASSESS-meso collaborative, Anna Bibby, Najib Rahman, Bethan Pugh, Liz Darlison, James Walters, Janet Fallon, John P Corcoran, Mairead Dixon, Kate Slaven, Avinash Aujayeb, Louise Brown, Paul Beckett, Kevin Blyth, Selina Tsim, John Maclay, Giles Cox, Paul Shaw, Naomi Chamberlin, Malcolm Lawson, Dipak Mukherjee, Gairin Dancey, Rafiqul Islam, Duneesha de Fonseka, Eleanor Mishra, Beth Sage, Charlotte Barr, Tony Pope, Sam Guglani, Saif Yousif, Andrew Stanton, Helen Day, Anna Morley, Kerra James, Sonia Patole, Emeka Azubuike-Dyer, Emma Tucker, Jenny Symonds, Nick Maskell, Rahul Bhatnagar, Steven Walker, David Arnold, Amelia Clive, Geraldine A Lynch, Jasmin Sadler-Ladell, Tine Panduro, Dawn Redwood, Tania Wainwright, Joanna Tilley, Christine Jones, Matthew Mills, Lyndsey Holt, Karl Jackson, Lorell Dismore, Leah Taylor, Helen Wallace, Mercy Korley, Zita Ibatuliniene, Alexandra Macpherson, Stephanie Hughes, Megan O’Hare, Charlene Dunne, Margaret Flynn, Susan Smith, Laurie Jones, Nadia Elkaram, Lesley Nichols, Debbie Shea, Kerry Goodsell, Lisa Hudig, Anna Roberson, Anglise Addison, Masuma Begum, Melissa Bagshaw, Hilary Thatcher, Sarah Cotter, Kate McRoberts

**Affiliations:** 1 Division of Clinical Medicine, School of Medicine and Population Health, University of Sheffield, Sheffield, UK; 2 Sheffield Teaching Hospitals NHS Foundation Trust, Sheffield, UK; 3 Clinical Trials Research Unit, Leeds Institute of Clinical Trials Research, University of Leeds, Leeds, UK; 4 University Hospitals of Leicester, Leicester, UK; 5 Institute of Genetics and Cancer, University of Edinburgh, Edinburgh, UK; 6 School of Allied Health Professionals, Nursing & Midwifery, University of Sheffield, Sheffield, UK; 7 Bristol Academic Respiratory Unit, University of Bristol, Bristol, UK

**Keywords:** Mesothelioma, Palliative care, Pain, Cohort study, Quality of life

## Abstract

**Background:**

Pain associated with malignant mesothelioma can be complex. The natural course of pain over the disease trajectory is not well studied. The aim of this study was to explore the prevalence, severity, impact and clinical course of pain for people with mesothelioma over a 12-month period.

**Methods:**

A prospective observational study was conducted as a component of a larger national, multi-centre cohort study on mesothelioma – ASSESS-meso (A prospective obServational cohort Study collecting data on dEmographics, Symptoms and biomarkerS in people with mesothelioma) (ISRCTN: 61,861,764). Adults (> = 18 years) newly diagnosed with mesothelioma were recruited from out-patient clinics in 20 hospitals. Baseline demographic and clinical data were recorded. Frequency, severity and impact of pain, quality of life and mood were collected using Patient-Reported Outcome Measures (e.g., EORTC Quality of Life Questionnaire Core Questionnaire PALliative Cancer [QLQ-C15-PAL], Brief Pain Inventory [BPI]). Follow-up occurred 3-monthly for 12-months.

**Results:**

Among the 150 participants recruited, mean age was 72.4 years (SD 8.1), with 112 (74.7%) being men. Baseline assessments showed a greater proportion of participants were affected by dyspnoea compared with pain. Overall, twenty-one (14.0%) participants reported moderate or severe chest pain (defined as > = 40/100) although none were receiving strong opioids. Median pain duration was 14.1 weeks (IQR 10–24.1). At 9–12 months, quality of life was poorer in those reporting moderate or severe pain (mean overall QLQ-C15-PAL score 41.7 [SD = 20.4] vs. 71.6 [SD = 19.5]) and this pain interfered more with daily activities (mean BPI interference index, 29.1 [SD = 19.1] vs. 14.4 [SD = 14.5]). Sixty-six (44.0%) participants died during the 12-months follow-up period.

**Conclusions:**

Participants reporting moderate or severe chest pain experienced poorer quality of life and none were receiving strong opioids. Given the limited prognosis, early identification of ‘high risk’ mesothelioma patients i.e. those who are most likely to have high symptom burden and poor prognosis, would help ensure timely input is given to implement pain management strategies. This includes Specialist Palliative Care guidance about opioid prescribing and interventional procedures.

Future research should be directed towards the optimum ways to assess and improve breathlessness, without neglecting the smaller group of people who experience severe pain.

**Supplementary Information:**

The online version contains supplementary material available at 10.1186/s12904-026-02187-w.

## Introduction

Malignant mesothelioma is an incurable, aggressive cancer with a globally variable incidence. Some of the highest rates (30 per million), however, are observed in the United Kingdom (UK) and Australia [1]. Global estimates have suggested there are 38,400 deaths per year from this [2]. Overall survival typically ranges from 8–12 months, although rarely, long-term survival is recognised [3]. Despite advances in treatment, prognosis remains poor, and patients with mesothelioma often experience debilitating symptoms, including pain and breathlessness [4], leading to a reduced quality of life. As a result, many have palliative care needs from an early stage.

In several cancers, there is growing evidence that early referral to Specialist Palliative Care teams can improve symptom control and quality of life [5, 6]. However, this benefit has not been demonstrated in mesothelioma. The RESPECT-Meso trial, conducted in the UK and Australia, randomised patients to either usual care or usual care with early Specialist Palliative Care input [7]. The trial did not show a significant difference in quality of life at 24 weeks [8]. It concluded that future research should focus on symptom-specific outcomes to better identify which patients would benefit most from Specialist Palliative Care and the optimal timing for intervention [8].

Pain is one of the most common symptoms in mesothelioma, usually arising in the chest wall [9]. It has multiple potential causes including local soft tissue infiltration with inflammation, bone destruction, intercostal and paravertebral nerve damage [10]. Various analgesics, including strong opioids, non-steroidal anti-inflammatory drugs, anti-convulsants, local anaesthetic agents and steroids are used. Despite these, pain control is often reported as sub-optimal [11]. Research has suggested benefit from radiotherapy in a selected cohort of patients [11], with ongoing studies comparing escalated versus standard dose radiotherapy and the impact on pain [12]. Interventional procedures e.g., percutaneous cervical cordotomy, have been shown to be effective and safe. They are, however, often undertaken late in the illness (average time being 1.3 months prior to death) [13].

By characterising the nature and impact of pain, it may be possible to identify patients who would benefit most from timely interventions, such as interventional pain procedures, or early Specialist Palliative Care engagement. Targeted, evidence-based approaches to pain management could then help optimise QoL and reduce the burden of suffering for patients with mesothelioma. Therefore, the primary aim of this study was to describe the prevalence, severity, impact and clinical course of pain for those newly diagnosed with mesothelioma over a 12-month period.

Specific objectives were to:

- Collect prospective, longitudinal data characterising pain over time.
- Explore the association between chest pain severity, quality of life and mood.

## Methods

### Study design

A prospective observational study was conducted as a component of a larger national, multi-centre cohort study on mesothelioma-ASSESS-meso (A prospective obServational cohort Study collecting data on dEmographics, Symptoms and biomarkerS in people with mesothelioma) (ISRCTN: 61,861,764). ASSESS-meso is a prospective observational study gathering data on demographics, symptoms, and biomarkers in individuals with mesothelioma. Part of its remit is to be a platform for future sub-studies focusing on different phenomena. This sub-study focused on pain was an example, funded after the start of ASSESS-Meso. Full protocol details are available [14]. Recruitment for ASSESS-meso began on July 4, 2017, and is ongoing, with a total of 572 participants enrolled as of September 2024. The study is being conducted at 20 hospitals across the UK (16 in England, two in Scotland, and two in Wales), and includes individuals newly diagnosed with mesothelioma at any anatomical site (i.e. within six weeks of diagnosis). ASSESS-meso collects Patient Reported Outcome Measures (PROMS) at baseline and during routine clinical follow-up appointments. Due to the COVID-19 pandemic, the initially planned 3-monthly follow-up assessments were adjusted to coincide with clinical appointments. Research nurses conducted assessments face-to-face, when possible, but virtual/telephone data collection was also used to maintain inclusivity and data collection continuity. Data was recorded in the online REDCap database. This sub-study analysed the frequency, severity and impact of pain experienced by participants over time.

### Participant recruitment

Informed consent was obtained from all participants. Participants were recruited from out-patient clinics following a diagnosis of mesothelioma. Eligibility criteria were:

### Inclusion criteria

- Multidisciplinary team-ratified diagnosis of mesothelioma (histological, cytological or clinico-radiological diagnosis)
- Able to provide fully informed consent
- Willing and able to comply with study follow-up assessments

### Exclusion criteria

- Age < 18 years old

### Data collection

This study collected demographic information including age, sex, living situation (alone or with others), postcode (used to determine socioeconomic status via the Index of Multiple Deprivation (IMD)), smoking and alcohol history, and ethnicity (collected from 1 st June 2022). Clinical data gathered included: date of mesothelioma diagnosis, disease site, histological type, staging [15], and asbestos exposure. Eastern Cooperative Oncology Group – Performance Status (ECOG-PS) [16], analgesic use including morphine milligram equivalent daily dose (MEDD) and any co-existing medical conditions were recorded.

At the baseline assessment, several PROMS were collected.

- Chest pain severity over the preceding 24-h was assessed using a 100 mm visual analogue scale (VAS), as the chest is the most common location for pain in mesothelioma [9].
- The Brief Pain Inventory (BPI) [17], a multi-dimensional pain assessment tool, was used to evaluate pain intensity and its impact on daily functioning (e.g. general activity, sleep, enjoyment of life and relationships).
- The EORTC Quality of Life Questionnaire Core Questionnaire PALliative Cancer (QLQ-C15-PAL), a shortened version of a questionnaire designed for palliative care patients, was used to assess symptoms and quality of life [18].
- Anxiety and depression were screened for using the Hospital Anxiety and Depression Scale (HADS) [19, 20].

Finally, baseline blood samples were taken to measure full blood count, creatinine, C-reactive protein (CRP), and serum mesothelin levels.

During follow-up visits, researchers collected data on chest pain severity (using the VAS), medication history, and whether the patient had received palliative radiotherapy or any interventional pain procedures. They also recorded any engagement with Specialist Palliative Care services, referral dates and type of team involved. The same baseline PROMS were also collected at each follow-up visit.

Data collection occurred in line with clinical scheduling, i.e. data was collected at routine clinical appointments with no additional research visits conducted. In most centres, this followed a 3-monthly schedule, however, the COVID-19 pandemic caused disruption to both clinical pathways and the availability of researchers to collect data, hence questionnaire completion did not occur on a regular schedule for all participants. This, alongside participants' deaths as time passed, resulted in a varying number of completed questionnaires at each pre-specified analysis timepoint. The number of completed questionnaires is reported for each questionnaire at each timepoint, as is the number of "missing" values, where a participant had filled in the questionnaire but not provided an answer for that specific question/data point.

### Cessation of follow-up

Follow-up assessments ceased if the participant died, withdrew from the sub-study or reached a 12-month follow-up period.

### Statistical analysis

As an exploratory sub-study, no formal sample size was calculated. We made the decision to conduct the analysis based on pre-specified 3-monthly timepoints rather than collating data from all ‘first follow-ups’ (regardless of whether this occurred at month 3 or month 5).

Patient demographics and pain characteristics were summarised using frequencies, percentages and means (standard deviations) and medians (interquartile ranges) where appropriate. Pain was classified moderate or severe if the VAS score was > = 40/100 at any point during follow up, and the time to first episode of moderate or severe chest pain was calculated.

PROMS were scored according to the relevant manuals and summarised overall, by time-point and by moderate or severe chest pain, versus no or mild chest pain groups. Data from participants who withdrew or died were included up until the point of exit from the study. Missing data are presented but not included in percentage calculations.

## Results

Over a 17-month period (1st July 2021—30th November 2022), 150 patients were recruited to ASSESS-meso and all consented to the sub-study. The twelve-month follow-up completed on 30th November 2023. The median length of follow-up was 36.9 weeks (IQR 5.4–43.1) (summary of follow-up in Supplementary Table 1).

### Baseline demographic and clinical details (Table 1)

**Table 1:** Baseline characteristics for those who did and did not report moderate or severe chest pain during follow-up

Characteristic	Presence of moderate or severe chest pain		Total (n = 150)
	Yes (n = 21)	No (n = 129)	
Sex			
Male	17 (81.0%)	95 (73.6%)	112 (74.7%)
Female	4 (19.0%)	34 (26.4%)	38 (25.3%)
Age			
Mean (S.D.) years	74.2 (7.9)	74.1 (8.2)	74.2 (8.1)
Ethnicity			
White: English, Welsh, Scottish, Northern Irish or British	15 (100.0%)	77 (97.5%)	92 (97.9%)
Other^1^	0 (0.0%)	2 (2.6%)	2 (2.6%)
Missing	6	50	56
Lives alone			
Yes	4 (19.0%)	25 (19.7%)	29 (19.6%)
No	17 (81.0%)	102 (80.3%)	119 (80.4%)
Missing	0	2	2
Smoking status			
Never smoker	8 (38.1%)	44 (35.2%)	52 (35.6%)
Current smoker	1 (4.8%)	10 (8.0%)	11 (7.5%)
Ex smoker	12 (57.1%)	71 (56.8%)	83 (56.8%)
Missing	0	4	4
Units of alcohol per week			
Mean (S.D.)	4.0 (6.4)	6.3 (12.0)	6.0 (11.4)
ECOG performance status			
0	2 (12.5%)	18 (21.4%)	20 (20.0%)
1	10 (62.5%)	52 (61.9%)	62 (62.0%)
2	3 (18.8%)	12 (14.3%)	15 (15.0%)
3	1 (6.3%)	2 (2.4%)	3 (3.0%)
4	0 (0.0%)	0 (0.0%)	0 (0.0%)
Missing	5	45	50
Index of multiple deprivation (IMD)			
1	1 (4.8%)	5 (3.9%)	6 (4.0%)
2	0 (0.0%)	10 (7.8%)	10 (6.7%)
3	1 (4.8%)	19 (14.8%)	20 (13.4%)
4	4 (19.0%)	14 (10.9%)	18 (12.1%)
5	2 (9.5%)	13 (10.2%)	15 (10.1%)
6	2 (9.5%)	11 (8.6%)	13 (8.7%)
7	0 (0.0%)	14 (10.9%)	14 (9.4%)
8	4 (19.0%)	11 (8.6%)	15 (10.1%)
9	5 (23.8%)	15 (11.7%)	20 (13.4%)
10	2 (9.5%)	16 (12.5%)	18 (12.1%)
Missing	0	1	1
Site of disease			
Pleural	19 (90.5%)	122 (94.6%)	141 (94.0%)
Peritoneal	0 (0.0%)	5 (3.9%)	5 (3.3%)
Pericardial	0 (0.0%)	1 (0.8%)	1 (0.7%)
Other	1 (4.8%)	1 (0.8%)	2 (1.3%)
Pleura and pericardium	1 (4.8%)	0 (0.0%)	1 (0.7%)
Basis of diagnosis			
Histological	18 (85.7%)	114 (88.4%)	132 (88.0%)
Cytological	2 (9.5%)	7 (5.4%)	9 (6.0%)
Clinico-radiological	1 (4.8%)	8 (6.2%)	9 (6.0%)
Histological diagnosis			
Epithelioid	14 (77.8%)	90 (79.6%)	104 (79.4%)
Sarcomatoid	1 (5.6%)	5 (4.4%)	6 (4.6%)
Biphasic	2 (11.1%)	11 (9.7%)	13 (9.9%)
Desmoplastic	0 (0.0%)	5 (4.4%)	5 (3.8%)
Other	1 (5.6%)	0 (0.0%)	1 (0.8%)
Unspecified	0 (0.0%)	2 (1.8%)	2 (1.5%)
Missing	0	1	1
Tumour staging			
1a	2 (14.3%)	23 (29.1%)	25 (26.9%)
1b	5 (35.7%)	11 (13.9%)	16 (17.2%)
2	1 (7.1%)	4 (5.1%)	5 (5.4%)
3a	1 (7.1%)	13 (16.5%)	14 (15.1%)
3b	4 (28.6%)	20 (25.3%)	24 (25.8%)
4	1 (7.1%)	8 (10.1%)	9 (9.7%)
Missing	7	50	57
Exposure to asbestos history			
No exposure recalled	5 (23.8%)	26 (20.2%)	31 (20.7%)
Environment exposure i.e. asbestos in walls/ceilings of home/workplace, but not disturbed	1 (4.8%)	23 (17.8%)	24 (16.0%)
Passive exposure i.e. worked in environment where others were working with asbestos. Dust/fibres were in the air, but participant was not working with it directly	3 (14.3%)	30 (23.3%)	33 (22.0%)
Active exposure i.e. participant was working directly with asbestos, generating dust or fibres	12 (57.1%)	50 (38.8%)	62 (41.3%)
Baseline blood tests (mean, S.D.)			
Haemoglobin (normal 120–175 g/l)	132.6 (20.21)	132.3 (20.62)	132.3 (20.50)
NLR^2^ (normal 1–2)	6.1 (5.50)	5.7 (4.80)	5.7 (4.88)
Platelets (normal 150–450 × 10^9^/l)	304.9 (97.28)	364.6 (165.40)	356.7 (159.1)
Creatinine (normal 45–104 µmol/L	81.8 (13.12)	79.9 (23.57)	80.1 (22.43)
CRP (normal < 5 mg/l)	48.2 (68.50)	40.3 (59.15)	41.3 (60.08)
Mesothelin (normal < 1.4 nM)	2.3 (1.98)	4.4 (6.25)	4.1 (5.91)

^1^Data have been grouped to into ‘Other’ to preserve anonymity

^2^NLR = neutrophil-to-lymphocyte ratio

Almost three-quarters (n = 112, 74.7%) of participants were men, and the mean age was 72.4 years (SD 8.1). Of the 94 participants with ethnicity data recorded, 92 self-identified as White-British (97.9%).

Almost half (n = 69, 46.3%) lived in more socio-economically deprived areas (IMD decile < = 5) and a fifth lived alone (n = 29, 19.6%). In the 100 participants who had a performance status recorded at baseline, 82 (82.0%) had a performance status 0–1. The majority of participants had pleural disease (n = 141, 94.0%) and where histologically confirmed (n = 132), most had epithelioid histology (n = 104, 79.4%). Asbestos exposure was recalled by 119 participants (79.3%), with the highest proportion describing direct exposure (n = 62, 41.3%). Overall baseline bloods showed participants had raised neutrophil-to-lymphocyte ratios, C-reactive protein and mesothelin levels.

At baseline, the most common symptom was breathlessness (n = 123, 82.0%); followed by cough (n = 48, 32.0%), weight loss (n = 46, 30.7%), chest pain (n = 40, 26.7%), and fatigue (n = 37, 24.7%). The most commonly reported co-morbidities were the presence of previous/other cancer (n = 28, 19.3%), asthma/COPD (n = 22, 14.7%), ischaemic heart disease (n = 16, 10.7%) and gastrointestinal conditions (n = 15, 10.0%).

### Deaths

Sixty-six (44.0%) participants died during the 12-months follow-up period. The median time between initial assessment and death was 5.4 months (IQR 2.4–8.7).

### Prevalence of moderate or severe pain

**Fig. 1:**
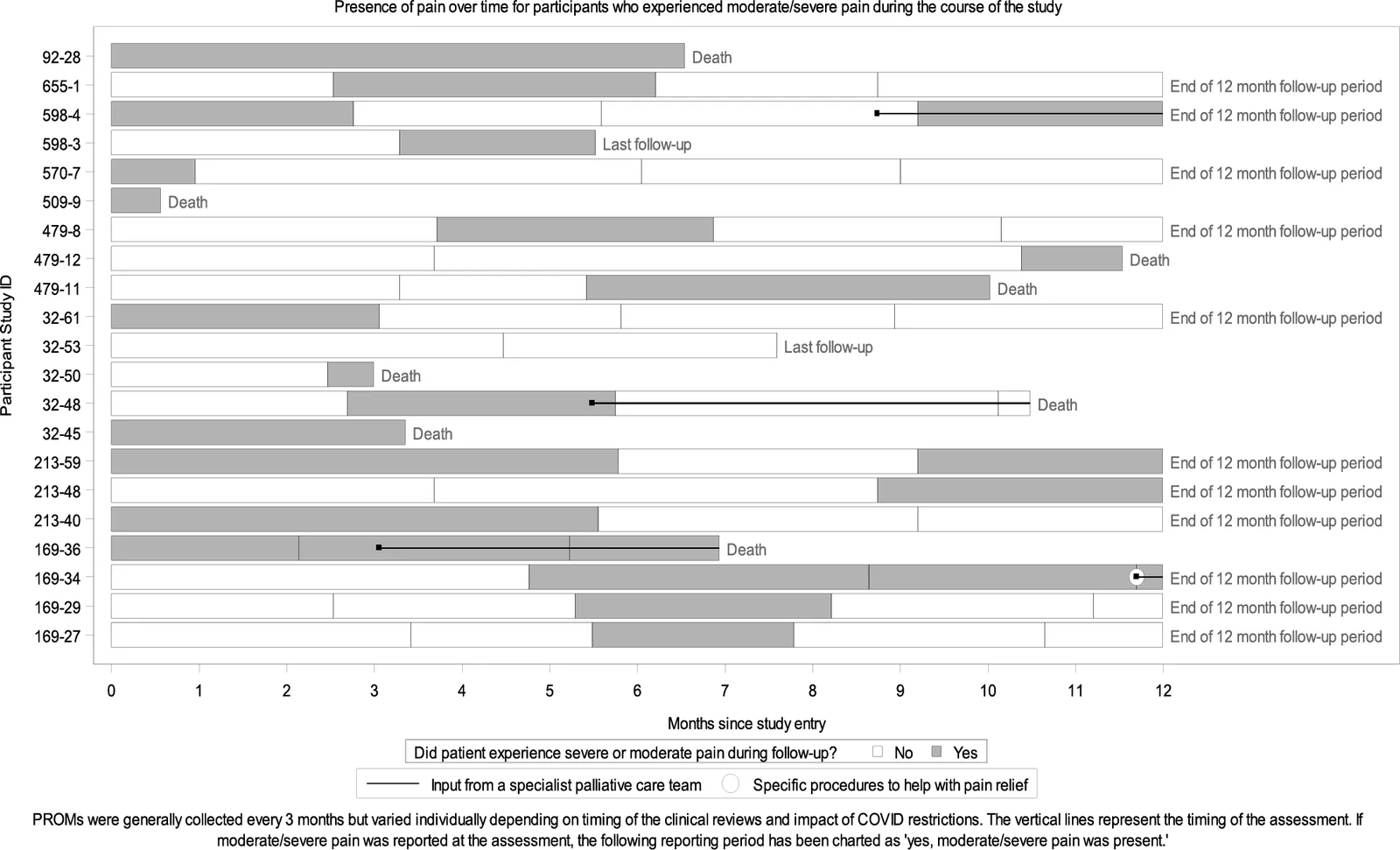
Swimmers plot demonstrating presence of pain over time for participants who reported moderate or severe chest pain during the study

Twenty-one (14.0%) participants reported moderate or severe chest pain during follow-up. Where pain was reported, median duration was 14.1 weeks (IQR 10–24.1). Those reporting moderate or severe chest pain were more frequently male (n = 17, 81.0% vs. n = 95, 73.6%) compared to those with mild or no chest pain (Table 1). Nine participants (42.9%) who reported moderate or severe chest pain had this at baseline. This pain fluctuated in severity over the disease trajectory (Fig. 1). Eight (38.1%) participants reporting moderate or severe chest pain died during follow-up and 7/8 (87.5%) reported this pain at their final assessment prior to death. In terms of analgesia, none of those experiencing moderate or severe chest pain were on strong opioids and three were on weak opioids. Additionally, none reported being on a non-steroidal anti-inflammatory drug (NSAID), an anti-depressant or an anti-epileptic adjuvant analgesia. For participants who did not report moderate or severe pain, ten were on strong opioids (MEDD 29.0 mg/24 h, S.D. 25.3) and nine were on weak opioids. Between two to five participants reported being on a non-steroidal anti-inflammatory drug (NSAID), an anti-depressant or an anti-epileptic adjuvant analgesia. Two participants (1.8%) reported receipt of a specific interventional pain procedure during their follow-up, only one of whom reported moderate or severe chest pain. Eight patients had palliative radiotherapy, three of whom reported moderate or severe chest pain.

Overall, sixteen participants (14.0%) received input from Specialist Palliative Care including community (n = 10) and hospital teams (n = 5); the team was not recorded for one participant. A greater proportion (n = 4/21, 22.2%) of participants reporting moderate or severe chest pain received Specialist Palliative Care input compared with those with no or mild pain (n = 12/129, 12.5%).

### BPI scores (Table 2)

**Table 2:** BPI interference scores for those who did and did not report moderate or severe chest pain during follow-up

	**Baseline**		**0–3 Months**		**3–6 Months**		**6–9 Months**		**9–12 Months**	
**Mean severity score**	**Pain**	**No pain**	**Pain**	**No pain**	**Pain**	**No pain**	**Pain**	**No pain**	**Pain**	**No pain**
Missing data^2^	1	0	0	0	0	0	0	0	0	0
N^1^	14	43	6	9	15	25	6	30	7	24
Mean (s.d.)	3.8 (1.7)	2.2 (1.6)	3.2 (1.3)	2.4 (2.3)	3.3 (1.8)	3.5 (2.1)	3.6 (0.6)	2.7 (1.4)	3.5 (1.8)	2.6 (1.7)
Median (range)	4.3 (0.8, 5.5)	2.0 (0.0, 7.0)	3.5 (1.5, 4.5)	1.5 (0.3, 6.5)	3.0 (1.0, 7.5)	3.0 (0.8, 7.0)	3.6 (2.5, 4.5)	2.8 (0.8, 5.3)	4.0 (1.0, 5.8)	2.4 (0.0, 7.3)
IQR	2.0, 5.3	0.8, 3.3	1.8, 4.3	0.8, 3.0	1.8, 4.8	2.0, 5.0	3.5, 3.8	1.5, 3.8	2.0, 5.3	1.5, 3.8

**Interference index**	**Pain**	**No pain**	**Pain**	**No pain**	**Pain**	**No pain**	**Pain**	**No pain**	**Pain**	**No pain**
N^1^	14	43	6	9	15	25	6	30	7	24
Mean (s.d.)	22.5 (18.6)	12.6 (15.6)	28.8 (16.1)	15.7 (17.5)	23.5 (16.0)	25.8 (20.3)	23.7 (18.9)	20.0 (19.4)	29.1 (19.1)	14.4 (14.5)
Median (range)	23.0 (1.0, 59.0)	7.0 (0.0, 61.0)	32.5 (0.0, 48.0)	12.0 (0.0, 51.0)	25.0 (3.0, 62.0)	26.0 (0.0, 68.0)	26.5 (0.0, 48.0)	13.5 (0.0, 54.0)	39.0 (0.0, 48.0)	7.5 (0.0, 54.0)
IQR	7.0, 37.0	0.0, 17.0	24.0, 36.0	0.0, 16.0	8.0, 32.0	7.0, 41.0	4.0, 37.0	5.0, 41.0	12.0, 45.0	4.5, 22.0

**Mean interference score**	**Pain**	**No pain**	**Pain**	**No pain**	**Pain**	**No pain**	**Pain**	**No pain**	**Pain**	**No pain**
N^1^	14	43	6	9	15	25	6	30	7	24
Mean (s.d.)	3.2 (2.7)	1.8 (2.3)	4.1 (2.3)	2.2 (2.5)	3.4 (2.3)	3.7 (2.9)	3.4 (2.7)	2.9 (2.8)	4.2 (2.7)	2.1 (2.1)
Median (range)	3.3 (0.1, 8.4)	1.0 (0.0, 8.7)	4.6 (0.0, 6.9)	1.7 (0.0, 7.3)	3.6 (0.4, 8.9)	3.7 (0.0, 9.7)	3.8 (0.0, 6.9)	1.9 (0.0, 7.7)	5.6 (0.0, 6.9)	1.1 (0.0, 7.7)
IQR	1.0, 5.3	0.0, 2.4	3.4, 5.1	0.0, 2.3	1.1, 5.3	1.0, 5.9	0.6, 5.3	0.7, 5.9	1.7, 6.4	0.6, 3.1

^1^*N* number of individuals answering ‘yes’ to question, ‘Throughout our lives, most of us have had pain from time to time (such as minor headaches, sprains, and toothaches). Have you had pain other than these everyday kinds of pain today?’; as data collection occurred around clinical schedules, not all participants completed questionnaires at each time point.

^2^Missing = questionnaire completed, but no answer provided for this individual question

Mean severity score has a range 0–10 and is the mean score of the 4 questions on pain severity with a higher score indicating higher severity of pain

The Interference index has possible ranges of 0–70 and is the sum of the questions on pain interference with a higher index indicating more interference

The Mean interference score has possible ranges of 0–10 and is the mean of the questions on pain interference with a higher score indicating more interference

Participants with moderate or severe chest pain reported higher mean severity scores and more interference with daily activities compared with those with no or mild pain at all timepoints except at 3–6-months. The latter difference was greatest at 9–12 months follow-up (mean BPI interference index, 29.1 [SD = 19.1] vs. 14.4 [SD = 14.5]).

In terms of ‘average’ pain scores in the previous 24 h reported on BPI, 33.3% (n = 19/57) participants with available data reported levels of 4/10 or above at baseline, compared with 40.0% (n = 16/40 participants at 3–6 months and 45.2% (n = 14/31) at 9–12 months (Supplementary Table 2).

### Quality of life (EORTC-C15-PAL) scores (Table 3)

**Table 3:** Comparison of EORTC QLQ-C15-PAL scores between those who did and did not report moderate or severe chest pain during follow-up

	**Baseline**		**0–3 Months**		**3–6 Months**		**6–9 Months**		**9–12 Months**	
**Pain**	Pain	No pain	Pain	No pain	Pain	No pain	Pain	No pain	Pain	No pain
Questionnaires completed	122		31		78		61		58	
N^1^	19	103	7	24	19	59	9	52	7	51
Missing^2^	0	0	0	0	0	0	0	0	0	0
Median (range)	33.3 (0.0, 83.3)	16.7 (0.0, 66.7)	50.0 (0.0, 83.3)	0.0 (0.0, 100)	33.3 (0.0, 66.7)	16.7 (0.0, 100)	16.7 (0.0, 83.3)	16.7 (0.0, 100)	50.0 (16.7, 100)	16.7 (0.0, 83.3)
IQR	16.7, 66.7	0.0, 33.3	16.7, 83.3	0.0, 25.0	16.7, 50.0	0.0, 33.3	0.0, 50.0	0.0, 33.3	16.7, 100	0.0, 33.3
Fatigue										
N^1^	17	102	7	24	19	58	9	52	6	51
Missing^2^	2	1	0	0	0	1	0	0	1	0
Median (range)	33.3 (0.0, 88.9)	33.3 (0.0, 100)	55.6 (22.2, 88.9)	22.2 (0.0, 100)	33.3 (22.2, 100)	33.3 (0.0, 100)	33.3 (22.2, 100)	33.3 (0.0, 100)	66.7 (0.0, 100)	33.3 (0.0, 100)
IQR	33.3, 55.6	22.2, 55.6	22.2, 88.9	0.0, 38.9	33.3, 66.7	33.3, 66.7	33.3, 44.4	22.2, 55.6	55.6, 88.9	22.2, 55.6
Dyspnoea										
N^1^	19	103	7	24	19	59	9	52	7	51
Missing^2^	0	0	0	0	0	0	0	0	0	0
Median (range)	33.3 (0.0, 100)	33.3 (0.0, 100)	66.7 (0.0, 66.7)	33.3 (0.0, 100)	33.3 (0.0, 100)	33.3 (0.0, 100)	33.3 (33.3, 100)	33.3 (0.0, 100)	33.3 (0.0, 100)	33.3 (0.0, 66.7)
IQR	33.3, 66.7	0.0, 33.3	0.0, 66.7	0.0, 50.0	33.3, 66.7	0.0, 33.3	33.3, 66.7	33.3, 33.3	0.0, 100	0.0, 33.3
Insomnia										
N^1^	19	102	7	24	19	59	9	52	7	51
Missing^2^	0	1	0	0	0	0	0	0	0	0
Median (range)	33.3 (0.0, 100)	33.3 (0.0, 100)	66.7 (0.0, 100)	16.7 (0.0, 100)	33.3 (0.0, 66.7)	33.3 (0.0, 100)	33.3 (0.0, 66.7)	33.3 (0.0, 100)	33.3 (0.0, 100)	33.3 (0.0, 100)
IQR	33.3, 66.7	0.0, 66.7	33.3, 100	0.0, 33.3	0.0, 66.7	0.0, 66.7	33.3, 33.3	0.0, 33.3	0.0, 66.7	0.0, 33.3
Appetite loss										
N^1^	19	103	7	24	19	59	9	52	7	51
Missing^2^	0	0	0	0	0	0	0	0	0	0
Median (range)	33.3 (0.0, 100)	33.3 (0.0, 100)	66.7 (0.0, 66.7)	0.0 (0.0, 100)	33.3 (0.0, 100)	33.3 (0.0, 100)	0.0 (0.0, 100)	0.0 (0.0, 100)	33.3 (0.0, 66.7)	0.0 (0.0, 100)
IQR	0.0, 66.7	0.0, 33.3	0.0, 66.7	0.0, 0.0	0.0, 66.7	0.0, 66.7	0.0, 33.3	0.0, 33.3	0.0, 66.7	0.0, 33.3
Constipation										
N^1^	17	103	7	24	19	59	9	52	6	51
Missing^2^	2	0	0	0	0	0	0	0	1	0
Median (range)	0.0 (0.0, 66.7)	0.0 (0.0, 100)	33.3 (0.0, 100)	0.0 (0.0, 66.7)	33.3 (0.0, 100)	33.3 (0.0, 100)	33.3 (0.0, 66.7)	0.0 (0.0, 100)	0.0 (0.0, 66.7)	0.0 (0.0, 100)
IQR	0.0, 33.3	0.0, 33.3	33.3, 66.7	0.0, 16.7	0.0, 66.7	0.0, 66.7	0.0, 33.3	0.0, 33.3	0.0, 0.0	0.0, 33.3
Nausea/vomiting										
N^1^	18	103	7	24	19	59	9	52	7	51
Missing^2^	1	0	0	0	0	0	0	0	0	0
Median (range)	0.0 (0.0, 100)	0.0 (0.0, 100)	16.7 (0.0, 50.0)	0.0 (0.0, 50.0)	0.0 (0.0, 50.0)	0.0 (0.0, 100)	0.0 (0.0, 0.0)	0.0 (0.0, 100)	0.0 (0.0, 50.0)	0.0 (0.0, 50.0)
IQR	0.0, 16.7	0.0, 16.7	0.0, 50.0	0.0, 8.3	0.0, 16.7	0.0, 16.7	0.0, 0.0	0.0, 8.3	0.0, 16.7	0.0, 0.0
Physical functioning										
N^1^	19	103	7	24	19	58	9	52	6	50
Missing^2^	0	0	0	0	0	1	0	0	1	1
Median (range)	73.3 (26.7, 93.3)	73.3 (20.0, 93.3)	60.0 (26.7, 73.3)	73.3 (0.0, 93.3)	73.3 (26.7, 93.3)	73.3 (6.7, 93.3)	73.3 (46.7, 93.3)	73.3 (0.0, 93.3)	53.3 (26.7, 93.3)	73.3 (13.3, 93.3)
IQR	60.0, 93.3	60.0, 93.3	46.7, 73.3	53.3, 93.3	46.7, 93.3	46.7, 93.3	60.0, 93.3	46.7, 93.3	26.7, 73.3	60.0, 93.3
Emotional functioning										
N^1^	17	103	7	24	19	59	9	52	6	51
Missing^2^	2	0	0	0	0	0	0	0	1	0
Median (range)	83.3 (0.0, 100)	83.3 (0.0, 100)	83.3 (41.7, 100)	100 (50.0, 100)	83.3 (41.7, 100)	83.3 (0.0, 100)	100 (66.7, 100)	100 (41.7, 100)	66.7 (16.7, 100)	83.3 (0.0, 100)
IQR	50.0, 100	66.7, 100	66.7, 100	83.3, 100	66.7, 100	66.7, 100	83.3, 100	75.0, 100	41.7, 100	66.7, 100
Overall quality of life (q30)										
N^1^	17	103	7	24	19	59	9	52	6	51
Missing^2^	2	0	0	0	0	0	0	0	1	0
Median (range)	66.7 (33.3, 100)	66.7 (16.7, 100)	66.7 (16.7, 83.3)	83.3 (16.7, 100)	66.7 (0.0, 83.3)	66.7 (16.7, 100)	66.7 (16.7, 83.3)	66.7 (0.0, 100)	33.3 (16.7, 66.7)	66.7 (33.3, 100)
IQR	33.3, 83.3	50.0, 83.3	50.0, 66.7	66.7, 83.3	33.3, 83.3	50.0, 83.3	50.0, 66.7	50.0, 83.3	33.3, 66.7	66.7, 83.3

^1^As data collection occurred around clinical schedules, not all participants completed questionnaires at each time point

^2^Missing = questionnaire completed, but no answer provided for this individual question

All the index possible ranges are 0–100; for Physical functioning and Emotional functioning, the higher the score the better the functioning, for the Overall quality of life, the higher the score the better the quality of life, and for all other indexes the higher the score the more prominent it is

Participants reporting moderate to severe chest pain reported higher symptom scores for pain, fatigue, dyspnoea, and appetite loss across the 12-month study period. Overall quality of life scores were consistently lower for those reporting moderate or severe chest pain compared with mild or no pain. The greatest difference in quality of life was seen at the 9–12 month timepoint (mean overall EORTC-C15-PAL score, 41.7 vs. 71.6 [SD = 20.4 vs. 19.5]), a difference greater than 10 points, which is generally regarded as both statistically and clinically significant [21].

### Anxiety and depression (Supplementary Table 3)

In total, 340 assessments were conducted using the HADs tool. Less than 10% recorded clinically relevant levels (defined as scores > 11/21) for anxiety (n = 33, 9.7%) or depression (n = 31, 9.1%). There were no consistent trends in anxiety and depression over time between those reporting moderate or severe chest pain and those who did not.

## Discussion

### Main findings

This prospective, multi-centre, UK-wide study of individuals with malignant mesothelioma revealed that a relatively small proportion of participants reported moderate or severe chest pain. A substantial proportion (one-third) of those experiencing chest pain, however, reported it at their final assessment prior to death, suggesting pain may escalate in the pre-terminal phase of the illness. Notably, none of these individuals reporting moderate or severe pain were receiving strong opioids. The use of Specialist Palliative Care teams was infrequent, and the application of interventional pain procedures was uncommon. Quality of life scores were lower in participants reporting moderate or severe chest pain and resulted in greater interference with daily functioning.

### What this study adds

This is the largest observational study focusing on pain in mesothelioma, and the only one to collect serial pain scores from a cohort representative of the UK population [22]. Compared with previous mesothelioma research, our findings suggest the prevalence of moderate or severe chest pain may be less common, although no less important when it occurs.

A post-hoc exploratory analysis of symptom burden from the RESPECT-Meso trial (n = 174) provides a relevant point of comparison [23]. In that trial, pain was assessed using the EORTC QLQ-C30, with additional symptom assessments conducted in the early Specialist Palliative Care arm (n = 87). RESPECT-Meso reported a 24% pain prevalence using the European Organisation for Research and Treatment of Cancer Quality of Life Questionnaire Core 30 (EORTC QLQ-C30). Higher rates were observed with the Edmonton Symptom Assessment Scale (ESAS) (60%) and Sheffield Profile for Assessment and Referral to Care tool (SPARC)(76%) tools [23]. Part of this variation may be due to the differing scoring systems for these tools i.e. SPARC has a 4-point Likert scale rating for pain from ‘not at all’ to ‘very much’ rather than a numerical scale which this study used. These additional symptom assessments were also only undertaken in the group receiving early Specialist Palliative Care rather than all participants.

Participants differed from those in our study as RESPECT-meso participants were required to have a performance status of 0 or 1 to be eligible. Additionally, anyone with ‘significant physical or psychiatric co-morbidity’, ‘high symptom burden at diagnosis’, already on systemic anticancer treatment or who had had recent thoracic surgery were excluded. These selection criteria limit the generalisability of RESPECT-meso’s findings to the wider mesothelioma population, in contrast to our study, which had broad eligibility criteria [14].

A high symptom burden was seen in RESPECT-meso with almost 70% of participants reporting at least three symptoms [23]. Both that study and ours highlighted dyspnoea as either the first or second most prevalent symptom, suggesting breathlessness may be more important than pain and needs additional clinical attention and research.

Our study also demonstrated lower levels of anxiety and depression than anticipated. A previous mesothelioma study, also using the HADs tool, reported levels of clinical depression and anxiety of over 30% and 50% respectively [24]. Those participants, however, were selected by convenience sampling. The sample had a high proportion of woman and postgraduate degree-holders suggesting likely selection bias and reduced generalisability of results. In RESPECT-meso, anxiety (collected using the SPARC tool) was the most common psychological symptom (47/86, 55.0%) [23]. Most (n = 35/47) reported a low severity level of anxiety and only four participants reported high levels of any psychological symptoms (low mood and effect on sex life). This correlates with the low levels of clinically significant anxiety and depression reported in our study.

We did not assess the psychological impact of mesothelioma on informal carers. Preliminary findings from other research suggest that carers experience more detrimental effects in terms of mood compared with patients [24]. In addition to patients’ feedback, routine collection of informal caregivers’ perceptions of pain and symptom control may provide additional useful insights.

There were low levels of involvement of Specialist Palliative Care teams, although referrals occurred more frequently when moderate or severe pain was present, highlighting that pain remains a main trigger for initiating referral. In contrast to a study by Temel [5], where early, routine Specialist Palliative Care referral for those diagnosed with metastatic non-small-cell lung cancer led to significant quality of life improvements, this was not the case for people affected by mesothelioma. Hence, it is not usual practice for mesothelioma patients to receive routine early Specialist Palliative Care input. Although pain can be a common symptom, the low levels of moderate or severe chest pain observed within our study may help explain this finding. Alternatively, this may reflect good palliative care support coming from existing multi-disciplinary care teams. In the UK, for example, Mesothelioma Clinical Nurse Specialists have a key role in providing and coordinating palliative care support and advocating on behalf of the patient and their family [25]. The low proportion of participants being on strong opioids potentially reflects an area of unmet need.

Deducing why there is a low proportion is difficult to clarify as there are no details or free-text notes within ASSESS-meso about the reasoning for commencing or stopping medication. Possible explanatory reasons include reluctance to prescribe opioids [26, 27] variability in pain severity, or the appropriate use of adjuvant analgesia. Alternatively, it may have been deemed the pain was not opioid responsive but there were knowledge gaps about appropriate alternative medications e.g., neuropathic agents to use. Patient-related factors may have also influenced opiate use e.g., stigma [28], stoicism or intolerable adverse effects. Alternatively, the developments of systemic anti-cancer treatments have symptomatic benefits including pain control which also may have influenced findings [29]. The reasoning for why those without pain were on opioids may be related to their use to control dyspnoea or that the medication used achieved good pain control.

Potentially, a more effective and resource-efficient approach would be to target Specialist Palliative Care to people most likely to benefit. Identifying ‘high risk’ patients e.g., those with increasing pain scores, poor performance status at presentation or more severe breathlessness, may be such prompts for immediate referral. Additionally, consideration as to who is best placed to support psychological symptom burden and advance care planning is important [4]. Further research about how Specialist Palliative Care can be most effective within mesothelioma care, given the results of this study as well as the RESPECT-meso trial, reflects an important area of exploration.

As some participants reported moderate or severe chest pain and died shortly afterwards, responsiveness to this symptom is pertinent. Internationally, assessment of holistic needs is recommended [30, 31] using specific tools which ‘trigger’ referral [32] or assess and guide timing of referral, for example SPARC [33]. Using the wider ASSESS-meso cohort to develop predictive prognostic modelling may provide further insight into who may benefit from Specialist Palliative Care.

### Strengths and limitations

A strength of this research is that these data come from the largest prospective, longitudinal cohort study in mesothelioma, ASSESS-meso, with a representative patient cohort [22].

Although our sample was predominately White British, this is in keeping with other studies e.g., a 10-year retrospective review of 247 mesothelioma patients in Northeast England (all participants were White British) [34].

The short life expectancy associated with mesothelioma meant that a large proportion of patients died before the end of the study follow-up period. The 12-month follow-up period was chosen so we could explore changes in pain scores in the pre-terminal phase. However, this meant that there were fewer data points available for analysis at later timepoints. Future studies would need to enrol larger number of participants over a longer time period to overcome this issue, but this emphasises the benefit of ongoing cohort studies such as ASSESS-meso.

We made the decision to conduct the analysis based on pre-specified 3-monthly timepoints rather than collating data from all ‘first follow-ups’. Unfortunately, the COVID-19 pandemic caused disruption to routine care, and study follow-up care was less regular as a result. This meant there was not a complete dataset for each timepoint, and especially low numbers at the 3-month assessment.

Finally, we don’t have detailed information about the composition and service provision of individual Specialist Palliative Care services for each site; hence the level of ‘Specialist Palliative Care involvement’ may vary.

## Conclusion

Although a relatively small proportion of participants reported moderate or severe chest pain, the impact on quality of life for those affected was substantial. Given the often-limited prognosis for mesothelioma, timely intervention for individuals experiencing significant pain is needed. Ensuring appropriate pain management strategies are implemented is fundamentally pertinent and may include interventional procedures. Specialist Palliative Care involvement should focus on providing guidance and reassurance regarding opioid prescribing as well as recommendations about additional interventions. Further research is needed to identify clinical markers and early warning symptoms of escalating pain, as well as to explore the use of holistic assessment tools to effectively prioritize patients for Specialist Palliative Care referral and intervention.

## Supplementary Information

Supplementary Material 1.

## Data Availability

The ASSESS-meso datasets used and/or analysed during the current study are available from Dr Anna Bibby, via [anna.bibby@bristol.ac.uk](mailto:anna.bibby@bristol.ac.uk) on reasonable request.

## Abbreviations

ASSESS-meso: A prospective obServational cohort Study collecting data on dEmographics, Symptoms and biomarkerS in people with mesothelioma
BPI: Brief Pain Inventory
CRP: C-reactive protein
ECOG-PS: Eastern Cooperative Oncology Group – Performance Status
HADS: Hospital Anxiety and Depression Scale
IMD: Index of Multiple Deprivation
IQR: Interquartile Range
MEDD: Morphine milligram equivalent daily dose
PROMs: Patient-Reported Outcome Measures
QLQ-C15-PAL: EORTC Quality of Life Questionnaire Core Questionnaire PALliative Cancer
SD: Standard Deviation
UK: United Kingdom
VAS: Visual Analogue Scale
