# Supplementary material for: Understanding pain in malignant mesothelioma: a prospective, longitudinal cohort study
Source: BMC Palliat Care. 2026 Jun 13;25:252. doi: 10.1186/s12904-026-02187-w (PMC13491870; doi:10.1186/s12904-026-02187-w)
Supplement: Supplementary file 1 [file 12904_2026_2187_MOESM1_ESM.docx]

**Supplementary Table 1. Summary of participant follow-up**

|  | **Baseline** | **0-3 Months** | **3-6 Months** | **6-9 Months** | **9-12 Months** |
| --- | --- | --- | --- | --- | --- |
| **Was participant followed up?** |  |  |  |  |  |
| Yes | 150 (100.0%) | 44 (29.3%) | 86 (57.3%) | 70 (46.7%) | 66 (44.0%) |
| No | 0 (0.0%) | 106 (70.7%) | 64 (42.7%) | 80 (53.3%) | 84 (56.0%) |
| Total | 150 (100%) | 150 (100%) | 150 (100%) | 150 (100%) | 150 (100%) |
| **Reason for no follow-up** |  |  |  |  |  |
| Death | - | 19 (17.9%) | 32 (50.0%) | 47 (58.8%) | 60 (71.4%) |
| No reason provided | - | 87 (82.1%) | 32 (50.0%) | 33 (41.3%) | 24 (28.6%) |
| Total | - | 106 (100%) | 64 (100%) | 80 (100%) | 84 (100%) |

Note: We made the decision to conduct the analysis based on pre-specified 3-monthly timepoints rather than collating data from all ‘first follow-ups’. Unfortunately, the COVID-19 pandemic caused disruption to routine care, and this meant there was not a complete dataset for each timepoint.

**Supplementary table 2. Individual items from the BPI – pain intensity**

| **Pain at its worst in the last 24 hours** | **Baseline** | **0-3 Months** | **3-6 Months** | **6-9 Months** | **9-12 Months** |
| --- | --- | --- | --- | --- | --- |
| 0 | 2 (3.4%) | 0 (0.0%) | 0 (0.0%) | 0 (0.0%) | 1 (3.2%) |
| 1 | 15 (25.9%) | 4 (26.7%) | 2 (5.0%) | 3 (8.3%) | 3 (9.7%) |
| 2 | 4 (6.9%) | 0 (0.0%) | 5 (12.5%) | 6 (16.7%) | 7 (22.6%) |
| 3 | 11 (19.0%) | 5 (33.3%) | 8 (20.0%) | 8 (22.2%) | 4 (12.9%) |
| 4 | 4 (6.9%) | 0 (0.0%) | 5 (12.5%) | 1 (2.8%) | 3 (9.7%) |
| 5 | 6 (10.3%) | 0 (0.0%) | 4 (10.0%) | 3 (8.3%) | 3 (9.7%) |
| 6 | 6 (10.3%) | 1 (6.7%) | 2 (5.0%) | 6 (16.7%) | 2 (6.5%) |
| 7 | 6 (10.3%) | 3 (20.0%) | 5 (12.5%) | 6 (16.7%) | 3 (9.7%) |
| 8 | 2 (3.4%) | 2 (13.3%) | 6 (15.0%) | 3 (8.3%) | 1 (3.2%) |
| 9 | 2 (3.4%) | 0 (0.0%) | 1 (2.5%) | 0 (0.0%) | 2 (6.5%) |
| 10 | 0 (0.0%) | 0 (0.0%) | 2 (5.0%) | 0 (0.0%) | 2 (6.5%) |
| Missing | 0 (0.0%) | 0 (0.0%) | 0 (0.0%) | 0 (0.0%) | 0 (0.0%) |
| Total | 58 (100%) | 15 (100%) | 40 (100%) | 36 (100%) | 31 (100%) |
| **Pain on average** | **Baseline** | **0-3 Months** | **3-6 Months** | **6-9 Months** | **9-12 Months** |
| 0 | 2 (3.4%) | 1 (6.7%) | 0 (0.0%) | 1 (2.8%) | 1 (3.2%) |
| 1 | 20 (34.5%) | 3 (20.0%) | 4 (10.0%) | 4 (11.1%) | 4 (12.9%) |
| 2 | 9 (15.5%) | 2 (13.3%) | 11 (27.5%) | 10 (27.8%) | 7 (22.6%) |
| 3 | 7 (12.1%) | 3 (20.0%) | 9 (22.5%) | 5 (13.9%) | 5 (16.1%) |
| 4 | 7 (12.1%) | 2 (13.3%) | 5 (12.5%) | 9 (25.0%) | 5 (16.1%) |
| 5 | 9 (15.5%) | 2 (13.3%) | 4 (10.0%) | 4 (11.1%) | 7 (22.6%) |
| 6 | 2 (3.4%) | 1 (6.7%) | 3 (7.5%) | 3 (8.3%) | 0 (0.0%) |
| 7 | 1 (1.7%) | 1 (6.7%) | 3 (7.5%) | 0 (0.0%) | 0 (0.0%) |
| 8 | 0 (0.0%) | 0 (0.0%) | 1 (2.5%) | 0 (0.0%) | 2 (6.5%) |
| 9 | 0 (0.0%) | 0 (0.0%) | 0 (0.0%) | 0 (0.0%) | 0 (0.0%) |
| 10 | 0 (0.0%) | 0 (0.0%) | 0 (0.0%) | 0 (0.0%) | 0 (0.0%) |
| Missing | 1 (1.7%) | 0 (0.0%) | 0 (0.0%) | 0 (0.0%) | 0 (0.0%) |
| Total | 58 (100%) | 15 (100%) | 40 (100%) | 36 (100%) | 31 (100%) |

Follow-up was disrupted by the COVID-19 pandemic and as a result some participants may have had multiple visits during a single timepoint i.e. during 0-3m follow-up. Summaries include all questionnaires completed by participants during each time-point.

**Supplementary Table 3. Summary of HADS Questionnaire scores by timepoint for those who did and did not report severe or moderate chest pain during follow-up.**

|  | **Baseline** | | **0-3 Months** | | **3-6 Months** | | **6-9 Months** | | **9-12 Months** | |
| --- | --- | --- | --- | --- | --- | --- | --- | --- | --- | --- |
|  | **Pain** | **No pain** | **Pain** | **No pain** | **Pain** | **No pain** | **Pain** | **No pain** | **Pain** | **No pain** |
| **Categorised HADS anxiety score** | | | | | | | | | | |
| Normal case | 12 (63.2%) | 68 (66.0%) | 5 (71.4%) | 19 (79.2%) | 17 (94.4%) | 37 (64.9%) | 9 (100.0%) | 37 (74.0%) | 5 (71.4%) | 33 (71.7%) |
| Borderline abnormal case | 4 (21.1%) | 21 (20.4%) | 2 (28.6%) | 4 (16.7%) | 1  (5.6%) | 11 (19.3%) | 0  (0.0%) | 8 (16.0%) | 1 (14.3%) | 8 (17.4%) |
| Abnormal case | 3 (15.8%) | 12 (11.7%) | 0  (0.0%) | 1  (4.2%) | 0  (0.0%) | 8 (14.0%) | 0  (0.0%) | 3  (6.0%) | 1 (14.3%) | 5 (10.9%) |
| Missing | 0  (0.0%) | 2  (1.9%) | 0  (0.0%) | 0  (0.0%) | 0  (0.0%) | 1  (1.8%) | 0  (0.0%) | 2  (4.0%) | 0  (0.0%) | 0  (0.0%) |
| Total | 19 (100%) | 103 (100%) | 7 (100%) | 24 (100%) | 18 (100%) | 57 (100%) | 9  (100%) | 50 (100%) | 7 (100%) | 46 (100%) |
| **Categorised HADS depression score** | | | | | | | | | | |
| Normal case | 15 (78.9%) | 84 (81.6%) | 6 (85.7%) | 20 (83.3%) | 15 (83.3%) | 39 (68.4%) | 5  (55.6%) | 40 (80.0%) | 4 (57.1%) | 32 (69.6%) |
| Borderline abnormal case | 3 (15.8%) | 12 (11.7%) | 1 (14.3%) | 2  (8.3%) | 0  (0.0%) | 10 (17.5%) | 2  (22.2%) | 4  (8.0%) | 2 (28.6%) | 8 (17.4%) |
| Abnormal case | 1  (5.3%) | 5  (4.9%) | 0  (0.0%) | 2  (8.3%) | 3 (16.7%) | 7 (12.3%) | 2  (22.2%) | 4  (8.0%) | 1 (14.3%) | 6 (13.0%) |
| Missing | 0  (0.0%) | 2  (1.9%) | 0  (0.0%) | 0  (0.0%) | 0  (0.0%) | 1  (1.8%) | 0  (0.0%) | 2  (4.0%) | 0  (0.0%) | 0  (0.0%) |
| Total | 19 (100%) | 103 (100%) | 7 (100%) | 24 (100%) | 18 (100%) | 57 (100%) | 9  (100%) | 50 (100%) | 7 (100%) | 46 (100%) |

Follow-up was disrupted by the COVID-19 pandemic and as a result some participants may have had multiple visits during a single timepoint i.e. during 0-3m follow-up. Summaries include all questionnaires completed by participants during each time-point.
